# P-408. Reducing Erroneous Penicillin Allergy Labels (REPeAL) in Pediatric Urgent Care Clinics

**DOI:** 10.1093/ofid/ofaf695.625

**Published:** 2026-01-11

**Authors:** Jennifer McKinsey, Rana E El Feghaly, Sheryl A Chadwick, Tiffany Addington, Holly B Austin, Maria Blanco, Emily J Montgomery, Amanda Nedved

**Affiliations:** Children's Mercy Kansas City, University of Missouri Kansas City School of Medicine, Overland Park, KS; Children's Mercy Kansas City, Kansas City, MO; Children's Mercy Kansas City, Kansas City, MO; Children's Mercy Kansas City, Kansas City, MO; Children's Mercy Hospitals and Clinics, Overland Park, Kansas; Children's Mercy Hospitals and Clinics, Overland Park, Kansas; Children's Mercy Kansas City, Kansas City, MO; Children's Mercy Kansas City; University of Missouri Kansas City School of Medicine, Lenexa, Kansas

## Abstract

**Background:**

10% of the US population reports a penicillin allergy label (PAL), yet only 1% has a true hypersensitivity. PALs are associated with worse health outcomes due to alternative broad-spectrum antibiotic use. We aimed to decrease the monthly percent of patients with PALs in our pediatric urgent care (UC) from 7.0% to 5.0% in 18 months.

**Figure:**
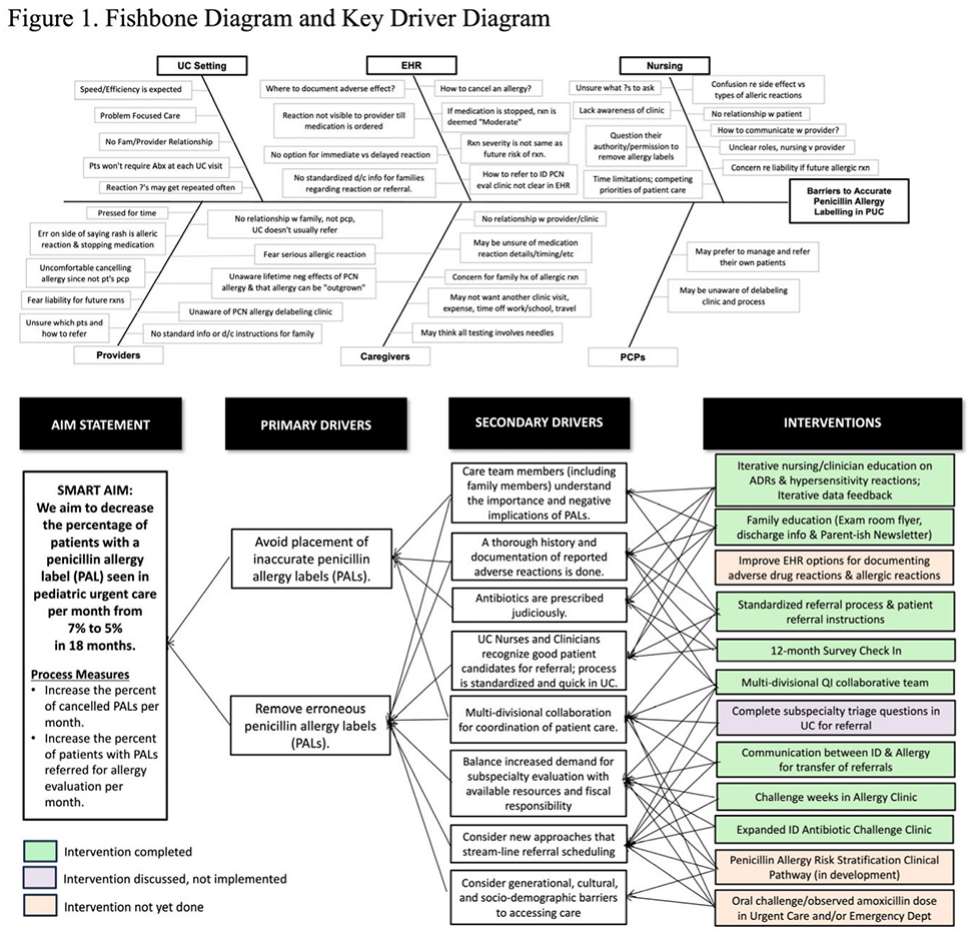

Urgent Care (UC), Electronic Health Record (EHR), Primary Care Providers (PCPs), Patients (Pts), Penicillin (PCN), Reaction (rxn), History (hx), Penicillin Allergy Label (PAL)

**Figure 2.**
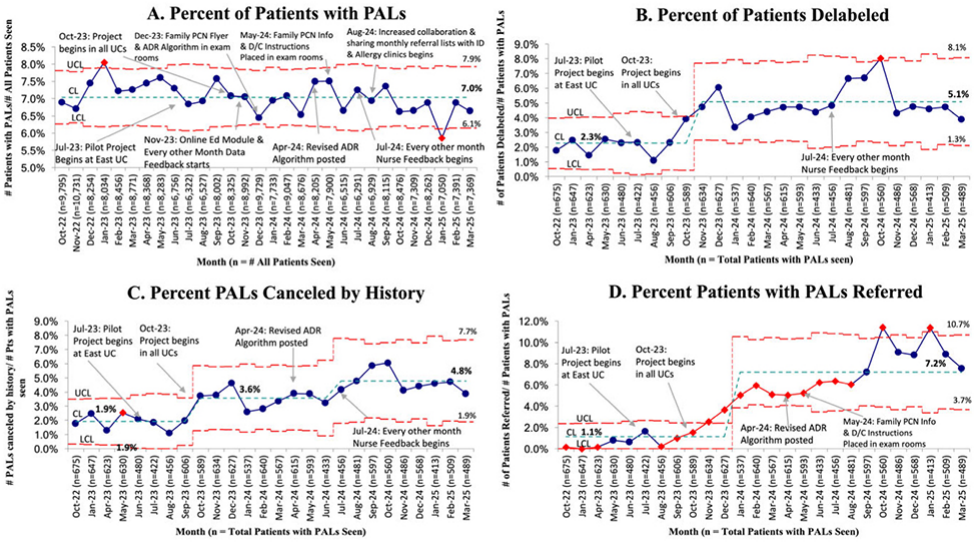
Shewhart Control Charts for (A) and (B) Outcome Measures and (C) and (D) Process Measures

**Methods:**

We used quality improvement methodology (Figure 1) to develop interventions which included education, feedback, an adverse drug reaction question algorithm, and a standardized subspecialty referral process. We reviewed UC encounters with PALs during baseline (Oct 2022-Sept 2023) and study periods (Oct 2023-Mar 2025). Monthly percent of patients with PALs and percent patients delabeled were our primary and secondary outcome measures. The monthly percent of PALs canceled by history alone and percent of patients referred for antibiotic challenge testing (ACT) were our process measures. The percent of patients who were relabeled with a PAL was our balancing measure. We used control charts to identify change over time. We surveyed UC staff after 12 months on their evaluation and management of PALs.

**Figure:**
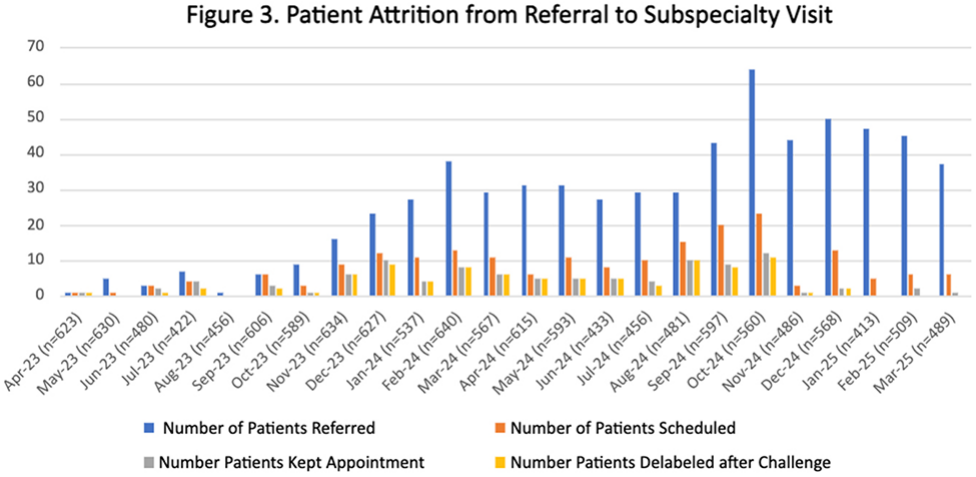

**Figure:**
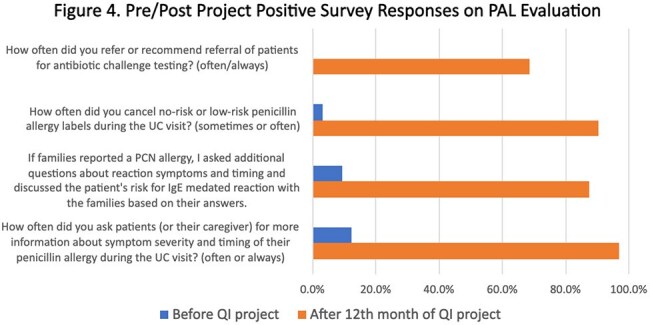

Penicillin Allergy Label (PAL), Penicillin (PCN), Quality Improvement (QI)

**Results:**

We reviewed 16,918 encounters. The center line (CL) for the percent of patients with PALs did not change (7.0%). The CL for the percent of patients delabeled had an upward shift from 2.3% to 5.1%. The percentage of PALs canceled had an upward CL shift from 1.9% to 4.8%. The percentage of patients referred for ACT had an upward CL shift from 1.1% to 7.2% (Figure 2). A total of 489 PALs were removed; 405 (83%) cancelled by history alone and 84 (17%) after ACT. Three delabeled patients had their PALs reapplied, however, none had hypersensitivity reactions. We observed significant patient attrition between referral and subspecialty visits (Figure 3). A 12-month survey showed increases in the percents of staff who reported they “often” or “always” request details about PALs (12.5% to 96.9%) and refer patients for allergy evaluation (0% to 68.8%) (Figure 4).

**Conclusion:**

Though we did not decrease the monthly percent of patients with PALs seen in our changing UC population, we saw a positive impact on our UC staff's approach to PALs, and we demonstrated that many patients can safely have their PALs canceled by taking a detailed history without subspecialty referral for ACT.

**Disclosures:**

Rana E. El Feghaly, MD, MSCI, CPHQ, Merck (Any division): Grant/Research Support|Pfizer: Honoraria|Pfizer: Grant review panel Amanda Nedved, M.D., Merck: Grant/Research Support

